# Efficacy of thyroid cyst sclerotherapy with polidocanol

**DOI:** 10.2478/raon-2026-0024

**Published:** 2026-05-14

**Authors:** Ziga Mercun, Laura Martinjak, Edvard Pirnat, Simona Gaberscek, Katja Zaletel, Katica Bajuk Studen

**Affiliations:** 1 Department of Internal Medicine, Faculty of Medicine, University of Ljubljana, Ljubljana, Slovenia; 2 Division of Nuclear Medicine, University Medical Centre Ljubljana, Ljubljana, Slovenia

**Keywords:** thyroid, cyst, sclerotherapy, polidocanol

## Abstract

**Background:**

Simple cysts are a common form of thyroid nodules. If the cyst is symptomatic, recurs after fine needle aspiration or increases in size, sclerotherapy of the cyst is indicated. Polidocanol can be used as a sclerosing agent, but there are few studies evaluating the efficacy of polidocanol in the sclerotherapy of thyroid cysts.

**Patients and methods:**

We conducted a clinical retrospective study in adult patients diagnosed with a simple thyroid cyst with largest diameter greater than 15 mm. Patients were treated with thyroid cyst sclerotherapy with polidocanol (group A, N = 36), ultrasound-guided fine-needle evacuation of the cyst (group B, N = 41) or they received no treatment and were managed only by follow-up (group C, N = 28). Thereafter, the size of the thyroid cyst at least 12 months after the baseline evaluation was recorded.

**Results:**

The three groups of patients did not differ significantly in terms of gender, thyrotropin level, thyroid antibody positivity or thyroglobulin level. However, they differed significantly in age (46.5 ± 15.8 years for group A, 51.2 ± 17.4 years for group B and 65.8 ± 13.7 years for group C, p < 0.001). The three patient groups differed significantly in the initial volume of cysts (20.1 ± 13.8 mL for group A, 15.0 ± 17.8 mL for group B and 4.2 ± 4.5 mL for group C, p < 0.001). In groups A and B, the final volume of the cyst was significantly lower than the initial volume (7.6 ± 12.1 mL, p < 0.001, and 6.1 ± 10.4 mL, p < 0.001, respectively), but not in group C (5.3 ± 7.9 mL). There was no significant difference in final cyst volume between the three groups (p = 0.764).

**Conclusions:**

The results of our study show that sclerotherapy of thyroid cysts with polidocanol is an effective and safe method for the treatment of large thyroid cysts. Currently, such treatment is more commonly used in younger patients.

## Introduction

Thyroid nodules are discrete lesions within the thyroid gland that are morphologically distinct from the surrounding thyroid parenchyma.^1,2^ Up to 60% of adults in the general population have one or more thyroid nodules. Simple cysts are a common form of thyroid nodule, accounting for 15–30% of thyroid nodules and are associated with a malignancy risk of virtually 0.^1^ Most thyroid cysts are small and therefore asymptomatic. However, as the cyst increases in size, patients often report discomfort due to pressure on the surrounding tissue or complain of cosmetic problems. The first choice for treatment of thyroid cysts is removal of the cyst fluid with fine needle aspiration, although recurrence is common with this method.^3^ The next option is treatment with a sclerosing agent, with ethanol being the most commonly reported in the literature.^4^ However, when sclerosing thyroid cysts with ethanol, adverse effects such as local pain and transient burning sense, dizziness and drunkenness, neck hematoma, transient hyperthyroidism, dysphonia, and others have been reported.^5–7^ Polidocanol is a liquid sclerosing agent that has been used as an effective alternative to ethanol for sclerotherapy of thyroid cysts since it is associated with less pain if spread to paranodular and subcutaneous tissue occurs, and lacks systemic side-effects such as alcohol intoxication.^8^

Since studies estimating polidocanol efficacy in thyroid cyst sclerotherapy are scarce^8–13^, the aim of this study was to estimate and report the efficacy of polidocanol sclerotherapy for the treatment of benign thyroid cysts in University Medical Centre Ljubljana.

## Patients and methods

We conducted a clinical study in adult patients diagnosed with a simple thyroid cyst with the largest diameter of more than 15 mm who were treated with thyroid cyst sclerotherapy with polidocanol at the Division of Nuclear Medicine of the University Medical Centre Ljubljana from 01.01.2013 to 31.12.2023 (group A). Patients treated with ultrasound-guided fine-needle evacuation of the cyst (group B), and patients who received no treatment and were followed up only (group C) were included as controls.

The study was approved by the National Medical Ethics Committee of the Republic of Slovenia (No. 0120-503/2023/3).

The patients’ medical records were analysed retrospectively.

Patients were divided into 3 groups:

− group A (treated with sclerotherapy with polidocanol): patients with simple thyroid cysts reporting compression symptoms or cosmetic concerns with a recurrence of the cyst after at least two ultrasound-guided fine-needle complete evacuations of the cyst;
− group B (treated with ultrasound-guided fineneedle evacuation of the cyst): patients with simple thyroid cysts reporting compression symptoms or cosmetic concerns;
− group C (follow-up only): patients with simple thyroid cysts diagnosed by thyroid ultrasound reporting no compression symptoms or cosmetic concerns.

For group A, all patients treated with sclerotherapy with polidocanol at the Division of Nuclear Medicine of the University Medical Centre Ljubljana from 01. 01. 2013 to 31. 12. 2023, were included. For groups B and C, patients were selected by retrospectively inspecting all medical records from 31. 12. 2023 backward to match the number of patients in group A.

Patients younger than 18 years of age, patients with solid or predominantly solid nodules containing a cystic component, or patients with incomplete baseline clinical, ultrasound (US) or laboratory data were excluded from the study.

The initial clinical parameters (age, gender), laboratory values and US parameters of the cyst (initial volume and largest diameter of the cyst) were recorded. The size of the thyroid cyst was then recorded at least 12 months after the baseline assessment (final volume and final largest diameter of the cyst). If the thyroid cyst size data were incomplete from the medical records after 12 months, patients were invited by telephone to come to our clinic for a follow-up visit and thyroid US to perform the measurement. For group A, medical records were additionally screened for possible side-effects of thyroid cyst sclerotherapy within the first week after the procedure and for possible recurrence of the thyroid cyst until last follow-up at the clinic.

### Thyroid ultrasound and sclerotherapy

Thyroid US and all procedures were performed on all patients by an experienced thyroid specialist using a 7.5 MHz US probe. The dimensions of the thyroid cyst were measured in mm (a, b, c) and the volume of the thyroid cyst was calculated in ml (formula: a x b x c x π/6).

In group A and B, under the guidance of ultrasound, an 18-gauge needle was inserted into the center of the cyst area. The cystic fluid was completely aspirated and the volume of the aspirated fluid recorded.

In group A, thyroid sclerotherapy with polidocanol was performed: after aspiration of the cyst, 3% polidocanol was injected into the cyst. If the volume of the aspirated fluid was < 10 mL, 2 mL of polidocanol were injected; if the volume of the aspirated fluid was 10–15 mL, 3 mL of polidocanol were injected, and if the volume of the aspirated fluid was > 15 mL, 4–5 mL of polidocanol were injected. Patients were asked to apply pressure over the puncture site for 15 minutes. The volume of polidocanol injected was recorded for each patient. One week after the sclerotherapy procedure, evacuation of the cyst was repeated if needed.

The complications of sclerotherapy were divided into minor (local pain, transient burning sense, neck hematoma, fever in the first week after procedure, increased heartbeat, dizziness, nausea and/or headache) and major (dysphonia with or without dysphagia, nodule infection, Horner’s syndrome, thyrotoxicosis or hypothyroidism, esophageal injury, tracheal injury).

### Laboratory analyses

The following laboratory parameters were analysed at baseline: thyrotropin (TSH), free thyroxine (fT_4_) and free triiodothyronine (fT_3_) levels, levels of antithyroglobulin (antiTg) and antithyroid peroxidase (antiTPO) antibodies (Ab), and thyroglobulin (Tg). All laboratory measurements were performed at the biochemical laboratory of the Division of Nuclear Medicine of the University Medical Centre Ljubljana. Serum concentrations of TSH, fT_4_, fT_3_, antiTPO and antiTg Ab were measured by ADVIA Centaur System (Siemens Medical Solutions Diagnostics). Tg was measured by Kryptor platform (Brahms), based on TRACETM (time-resolved amplified cryptate emission) method. The levels of antiTg and antiTPO Ab were only recorded as pathological (positive) or non-pathological (negative), as the methods and units for determining the antibodies varied in the different time periods.

### Statistical analysis

The statistical analysis was performed with PSPP, version 2.0.1. Microsoft Excel, version 16.84, was used for data collection, basic volume calculations, volume changes and graphical representations. Data are presented as mean ± standard deviation (range).

Nominal difference in thyroid cyst volume was calculated as the difference between final and baseline thyroid cyst volume. Relative difference was the nominal difference divided by baseline volume. A clinically significant change in cyst size was defined as an increase or decrease in cyst volume of at least 50%, according to the European Thyroid Association (ETA) criteria for thyroid nodules.^4^

Continuous variables (age, TSH, fT_4_, fT_3_, Tg concentration, baseline and final cyst volume) were compared using a parametric (one-way ANOVA) and a non-parametric test (Kruskal-Wallis). The Bonferroni correction was used. A paired t-test was used to analyse the data within groups (polidocanol volume for cyst sclerotherapy in group A, nominal difference in cyst volume). A two-sample t-test was used when comparing two groups (relative change in cyst volume in participants categorised in group A or B or by gender). A chi-square test was used to test the significance of categorical variables (pathological levels antiTg and antiTPO Ab, clinically significant changes in cyst volume and cyst size). When continuous outcomes were tested for their dependence on continuous variables, linear regression was used (e.g., volume of polidocanol used per cyst versus initial cyst volume; relative change in cyst volume versus patient age), as well as Pearson correlation (for the association between relative change in cyst volume and patient age). A p-value below 0.05 was considered statistically significant.

## Results

The basic characteristics of the patients included in the study are listed in Table 1.

**TABLE 1.** Demographic characteristics of the patients and laboratory parameters at the first examination

Group	A (N = 36)	B (N = 41)	C (N = 28)	P
Age [years]	46.5 ± 15.8	51.2 ± 17.4	65.8 ± 13.7	**< 0.001**
Sex [F/M; % female]	20/16 (56%)	28/13 (68%)	21/7 (75%)	0.33
TSH [mIU/L]	1.59 ± 1.0	1.61 ± 0.9	2.46 ± 2.5	0.43
fT_4_ [pmol/L]	15.14 ± 1.9	15.22 ± 2.2	14.44 ± 2.9	0.33
fT_3_ [pmol/L]	5.06 ± 0.6	5.24 ± 0.4	4.97 ± 0.5	**0.04**
Positive antiTg antibodies [%]	17.2	5.6	12.0	0.07
Positive antiTPO antibodies [%]	12.5	2.8	8.0	0.32
Tg [μg/L]	61.55 ± 127.4	493.92 ± 2020.0	26.13 ± 25.15	0.30

1 antiTg = antithyroglobulin; antiTPO = antithyroid peroxidase; fT_3_ = free triiodothyronine; fT_4_ = free thyroxine; TSH = thyrotropin (TSH)

The time from initial examination to final evaluation was 31.5 ± 29 months in group A, 20.2 ± 9.6 months in group B and 18.0 ± 8.5 months in group C. The volume of evacuated fluid from the cyst was 19.3 ± 16.1 mL in group A and 15.4 ± 19.8 mL in group B. The amount of polidocanol used for cyst sclerotherapy in group A ranged from 2 to 5 mL, with a mean of 3.1 ± 1.0 mL.

**FIGURE 1.**
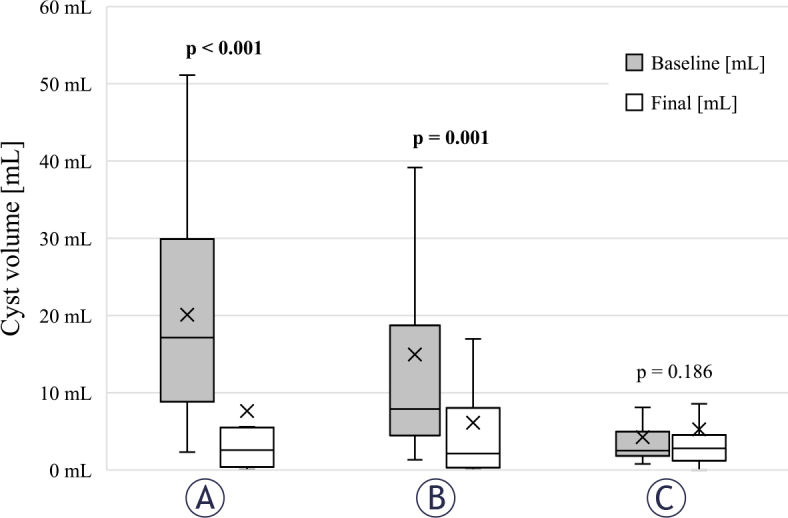
Initial and final volume of cysts in three groups of patients with simple thyroid cysts (group **(A)** – treated with thyroid sclerotherapy with polidocanol; group **(B)** – treated with ultrasound-guided fine-needle evacuation of the cyst, group **(C)** – no treatment; x represents mean initial and final cyst volume for each group).

Figure 1 and Table 2 show the initial and final cyst volume, the initial and largest cyst diameter as well as the nominal and relative change in cyst volume. Table 3 shows the percentage and absolute number of cysts with a clinically significant or insignificant change in size according to the ETA criteria.^4^

**TABLE 2.** Initial and final cyst volume, initial and final largest cyst diameter, and relative and absolute change in cyst volume

Group	A	B	C	p (A:B:C)	p (A:B)	p (A:C)	p (B:C)
Initial cyst volume [mL]	20.1 ± 13.8 (2.3-51.1)	15.0 ± 17.8 (1.3-83.0)	4.2 ± 4.5 (0,8-19,2)	**< 0.001**	**–**	**–**	**–**
Initial largest cyst diameter [mm]	41.6 ± 11.4 (23-62)	34.4 ± 12.8 (18-65)	23.4 ± 6.6 (15-47)	**< 0.001**	**–**	**–**	**–**
Final cyst volume [mL]	7.6 ± 12.1 (3.6-11.7)	6.1 ± 10.4 (2.8-9.4)	5.3 ± 7.9 (2.2-8.3)	0.764	**–**	**–**	**–**
Final largest cyst diameter [mm]	24.5 ± 15.4 (5-56)	21.5 ± 14.3 (4-65)	23.2 ± 11.6 (0-55)	0.491	**–**	**–**	**–**
Average relative cyst volume difference [%]	^a^-63.5 ± 45.5 (-78.9 to -48.2)	-48.2 ± 81.0 (-73.8 to -22.6)	8.1 ± 63.6 (-16.6 to 32.8)	**< 0.001**	0.318	**< 0.001**	**0.002**
Average nominal cyst volume difference [mL]	^a^12.5 ± 14.7 (7.5-17.4)	^b^8.83 ± 15.6 (3.9-13.7)	^c^-1.0 ± 4.0 (-2.5 to 0.52)	**< 0.001**	**–**	**–**	**–**

a p < 0.001 when compared with initial volume

b p = 0.001 when compared with initial volume

c p = 0.186 when compared with initial volume

**TABLE 3.** Percentage and absolute number of cysts with a clinically significant change in size according to the criteria of the European Thyroid Association (at least 50% increase or decrease in cyst volume or at least 20% increase or decrease in at least two cyst dimensions).^4^

Group	A	B	C	p (A:B:C)	p (A:B)	p (A:C)	p (B:C)
Significant decrease of cyst volume (%, absolute number)	75.0 (27/36)	68.3 (28/41)	25.0 (7/28)	**< 0.001**	0.616	**< 0.001**	**0.001**
Significant increase of cyst volume (%, absolute number)	2.8 (1/36)	7.3 (3/41)	39.2 (11/28)	**< 0.001**	**–**	**–**	**–**
No significant change in cyst volume (%, absolute number)	22.2 (8/36)	24.3 (10/41)	35.7 (10/28)	**–**	**–**	**–**	**–**

In group A, the rate of clinically significant change in size of thyroid cysts was not significantly influenced by the initial evacuation volume (p = 0.79). Gender (p = 0.068) and sex (p = 0.271) were not correlated with the relative change in cyst volumes.

In 1 (2.8%) patient in group A, re-treatment with sclerotherapy was performed after one year due to the recurrence of the cyst. After 9 years of followup, there was no recurrence of the cyst in that patient. In group A, 3 (8.3%) patients were referred to thyroidectomy due to the recurrence of the cyst (1 patient after 4 months, 1 patient after 1 year and 1 patient after 3 years since sclerotherapy).

Regarding side effects in group A during the procedure or within the first week after sclerotherapy, local pain was recorded in 8 (22.2%), fever in 7 (19.4%), and nausea and vomiting in 1 (2.8%) patient. No major side effects were recorded in this group.

## Discussion

In our study, we report single-centre results on the efficacy of sclerotherapy of thyroid cysts with polidocanol. We were able to confirm that at least 12 months after sclerotherapy with polidocanol, 75% of simple thyroid cysts were significantly smaller according to the criteria of the ETA.^4^ Thyroid cysts in group A, where sclerotherapy was indicated due to the recurrence of the cyst after at least two simple aspirations of the cyst, and group B, where only one evacuation of the cyst was needed, were initially significantly larger than the cysts in group C, where no treatment was indicated. In this study, we were able to show that after treatment, the volume of thyroid cysts in groups A and B was not significantly different from that of control group C, further emphasizing the efficacy of our approach to the treatment of thyroid cysts.

In our present study, the rate of significant (at least 50%) reduction in the volume of thyroid cysts with polidocanol sclerotherapy was 75%. A study by Gao *et al*. reported a significant reduction of the volume in 80.4% of thyroid cysts at 12 months; however, the mean baseline volume of the cysts in that study was smaller (14.65 mL) and the volume of lauromacrogol (polidocanol) injected corresponded to approximately 30% of the aspirated fluid.^12^ Surprisingly, a study by Chen *et al*. reported a reduction of volume of 83.51% of the thyroid cysts at 3 moths with only 2 mL of polidocanol injected; the baseline cyst volume was 24.76 mL.^13^ Gong *et al*. reported a 12-month therapeutic success of 98.67% with the baseline cyst volume of 14.5 mL and with the amount of injected polidocanol representing 25–35% of the aspirated fluid.^8^ Dong *et al*. reported a 73.2% effective treatment rate; however, they included predominantly cystic nodules with a mean volume of 12.5 mL, with a 12% regrowth rate at last follow-up.^12^ In that study, the volume of injected polidocanol represented 30–50% of the volume of the extracted fluid. Due to the differences in the baseline volume of the cysts and the volume of polidocanol injected, the comparison among the studies is difficult. However, Chen *et al*. suggested that not the volume of the sclerosant but a smaller initial volume of the cyst provides a larger relative contact area between the sclerosing agent and the cyst wall, enhancing the ablative effect.^13^ Clearly, a prospective randomized study with a larger number of patients is warranted to provide an appropriate initial volume of the cyst and the volume of polidocanol to be injected for optimum results. In addition, the use of ultrasound-guided microwave ablation (MWA) in combination with polidocanol injection has been reported as an effective approach for the treatment of benign cystic solid thyroid nodules.^14^ Another study compared the therapeutic efficacy and safety of MWA or polidocanol injection in cystic thyroid nodules and reported that both are very effective and safe treatment options; however, polidocanol was found to be more beneficial as it is less expensive and associated with a shorter hospital stay than MWA.^15^

Although sclerotherapy of thyroid cysts with ethanol is listed in the guidelines, the use of this sclerosing agent has been reported to be associated with adverse effects as it can leak outside the thyroid capsule.^5,7^ Scappaticcio *et al*. reported a 32% rate of complications of thyroid cyst sclerotherapy with ethanol, with the prevalence of minor complications of 32% (local pain, transient burning sense, neck hematoma, dizziness/drunkenness, flushing, increased heartbeat, nausea, headache, fever, vasovagal reaction), and major complications of 2% (dysphonia, dysphagia, arrhythmia, nodule infection, Horner’s syndrome, thyrotoxicosis, hypothyroidism, oesophageal injury, tracheal injury, skin burning).^7^ Therefore, polidocanol represents an effective alternative since it is associated with less pain if spread to paranodular and subcutaneous tissue occurs, and lacks systemic side-effects such as alcohol intoxication.^8^ In our study, local pain was reported by 22.2% patients within the first week after procedure, which is more than other studies with polidocanol but still significantly less frequent than with ethanol sclerotherapy. We report no major complications of polidocanol sclerotherapy, similarly to other studies with polidocanol, which confirms a better safety profile of polidocanol.^8–13^

Polidocanol is an effective alternative sclerosant to ethanol for thyroid cyst sclerotherapy, consisting of 95% hydroxypolyethoxydodedecane and 5% ethyl alcohol and is authorised as a medical sclerosant in European countries and also in the USA.^10^ The mechanism for treating the cyst is probably the destruction of the endothelial cells of the capsule wall, which leads to aseptic inflammation; this causes the endothelial tissue to atrophy and the walls of the cyst cavity to stick together and occlude.^10^ Histopathological evaluation of the process in a rabbit model showed that inflammation and fibrosis increased significantly, suggesting that polidocanol can be used effectively to treat thyroid nodules due to fibrosis.^16^ It also has an anaesthetic effect, so no local anaesthesia of patients is required.^8^ However, the most important clinical application of polidocanol is its endovascular use for the sclerotherapy of varicose veins.^17^

In our study, we report a significant reduction of the size of the cyst both after simple evacuation of the cyst and sclerotherapy. Of note, patients were referred to sclerotherapy only if the cyst recurred after at least two complete simple evacuations. Very little is known about the factors that lead to a less favourable outcome in large thyroid cysts that eventually require sclerotherapy and should be therefore the subject of further investigation.^18^ In our study, there was a clinically significant difference in age and free T_3_ levels between the three groups, with age being lower and free T_3_ levels being higher in the group A. However, as this was a clinical retrospective study without randomization, we believe that this is likely due to the fact that patients selected for polidocanol sclerotherapy, which is a more invasive procedure than simple aspiration, were more often younger. Therefore, the higher free T_3_ levels most likely reflect a lower age.^19^

The present study has several limitations. One of these is the clinical nature of the study, which did not allow randomization of the patients. Another limitation of the study was its retrospective nature. As it was a single centre study, the number of patients included is relatively small, although all data on sclerotherapy of thyroid cysts were collected over a 10-year period at our centre. This raises the issue of potential bias of patient inclusion in the study. However, as data on the efficacy of polidocanol sclerotherapy for thyroid cysts is very scarce in the scientific literature^8–13^, we believe that the data we report will contribute to a more frequent clinical use of this sclerosing agent. The advantage of our study in comparison to other published studies is the inclusion of two clearly defined control groups with thyroid cysts that required simple evacuation of the cyst only or no treatment. In other studies, the inclusion criteria for thyroid cyst sclerotherapy are stated only as a thyroid cyst causing compression or cosmetic concerns (no report of prior simple evacuation of the cyst or size for inclusion).

In conclusion, the results of our study show that sclerotherapy of recurrent thyroid cysts with polidocanol is an effective and safe method of treatment. Further prospective randomized studies including a larger number of patients are needed to confirm our data and define the appropriate volume of polidocanol to be injected for optimum results.
